# Cellular and humoral immune response to recombinant *Escherichia coli* OmpA in cows

**DOI:** 10.1371/journal.pone.0187369

**Published:** 2017-10-31

**Authors:** Pascal Rainard, Maryline Répérant-Ferter, Christophe Gitton, Florence B. Gilbert, Pierre Germon

**Affiliations:** Infectiologie et Santé Publique, UMR 1282, INRA, Université Tours, Nouzilly, France; Instituto Butantan, BRAZIL

## Abstract

The outer membrane protein (Omp) A is a major constituent of the outer membrane of *Escherichia coli*. This protein has been used in several vaccine development studies, but seldom with a view to vaccinating against mastitis. The objective of this study was to investigate the immunogenicity of *E*. *coli* OmpA and its vaccine potential for cows. Both the humoral and cellular immune responses were investigated. The gene for OmpA of the mastitis-causing strain P4 was cloned and expressed, and the recombinant protein (rEcOmpA) purified. Cows were immunized twice with rEcOmpA with adjuvant one month apart by the systemic route. Before immunization, few antibodies to rEcOmpA were detected, and there was little production of IL-17A in a whole blood stimulation assay (WBA) with rEcOmpA. Antibodies to rEcOmpA were induced by immunization. These antibodies were not able to react with *E*. *coli* P4, but reacted with a rough P4 mutant prepared by inactivating the *rfb* locus. This suggests that the complete LPS O-chain precluded the accessibility of antibodies to their target at the outer membrane. The cellular immune response appeared to be biased towards a Th17-type, as more IL-17A than IFN-γ was produced in the OmpA-specific WBA. There was a good correlation between antibody titers and the production of IL-17A in the WBA. The intramammary instillation of rEcOmpA elicited a slight local inflammatory response which was not related to the WBA. Overall, the interest of OmpA as vaccine immunogen was not established, although other experimental conditions (dose, adjuvant, route) need to be investigated to conclude definitively. The study pointed to several important issues such as the accessibility of OmpA to antibodies and the weakness of Th1-type response induced by OmpA.

## Introduction

The outer membrane protein A (OmpA) of *Escherichia coli* is an abundant integral protein of the outer membrane, which occurs at about 100,000 copies per cell [1]. As a transmembrane porin, OmpA plays a structural role in the integrity of the bacterial cell surface, and has multiple physiological functions. In relation to its exposure at the surface of the outer membrane, the OmpA protein is the target of several bacteriocins and bacteriophages and plays versatile roles in infection [2]. It has been shown to interfere with complement activation and to interact with immune cells, leading to decreased complement-dependent or phagocytic-dependent killing of OmpA-positive *E*. *coli* [3]. For these reasons and because it belongs to a class of bacterial proteins highly conserved among the *Enterobacteriaceae* family, OmpA has been considered as a potential antigen in vaccine research [4, 5]. The protein OmpA was considered as a target for host adaptive immune responses, specifically those directed to the loops of the molecule exposed at the surface of the outer membrane [6]. In spite of the importance of *E*. *coli* mastitis in dairy farming and an active research on mastitis vaccine, OmpA has seldom been considered as a potential vaccine antigen to induce protection against *E*. *coli* udder infections [7]. Consequently, although OmpA proved to be immunogenic in mice [5], limited information on its immunogenicity is available for the bovine species. In this study, we investigated the immunogenicity of *E*. *coli* OmpA and its vaccine potential for cows. We examined the antibody response to recombinant *E*. *coli* OmpA (rEcOmpA), paying attention to the capacity of antibodies to react with bacterial bodies. In addition, we examined the cell-mediated immune response to rEcOmpA by measuring the production of IL-17A and IFN-γ in an antigen-specific whole blood assay (WBA). We had previously shown that this assay can be used as a predictive indicator of the sensitization of the mammary gland to the antigen and that it involves the participation of CD4+ T cells [8]. Instillation of the sensitizing antigen into the lumen of the mammary gland induces a neutrophilic inflammation characterized by a massive influx of neutrophils into the milk. As the early recruitment of neutrophils into the mammary gland constitutes a major defense against infection [9], and because the antigen-specific and innate immune responses cooperate to amplify milk leukocytosis [10], this cell-mediated immune response can be considered as a useful feature for a potential vaccine antigen. Recently, we have shown that IL-17A is involved in the defense of the mammary gland against *E*. *coli*, most likely through the induction of neutrophilic inflammation and the amplification of the response of bovine mammary epithelial cells to *E*. *coli* [11, 12]. Consequently, and because porins of certain Gram negative bacteria exert a polarizing immunomodulatory effect [13], we paid attention to the Th1/Th17 balance of the immune response to rEcOmpA.

## Materials and methods

### Ethics statement

All animal experiments were conducted at the Teaching and Research herd of the LEGTA (Domaines d’Areines, Vendôme, France) with the approval of the ethics committee “Comité d’éthique pour l’expérimentation animale du Val de Loire” (agreement No. 2012-10-12 V2). Animal studies were compliant with all applicable provisions established by the European directive 2010/63/UE.

### *Escherichia coli* strain and mutant used in the study

Strain P4 is a prototypical *E*. *coli* strain (O32:H37) of bovine origin isolated from a case of clinical mastitis [14]. From this strain a rough mutant was prepared by disabling the *rfb* gene cluster, thereby preventing the synthesis of the O antigen. The *rfb* cluster of strain P4 was deleted using the method described by Datsenko and Wanner [15]. Primers used for deletion of the entire *rfb* cluster were PG512 and PG513 (Table 1). Deletion of the *rfb* cluster was confirmed using primers PG514 and PG515.

**Table 1:** List of primers used in this study.

Primer name	Primer sequence^a^
Deletion of rfb cluster	
PG512	CAGGTAGCTGTTGAGCTTGGGGCGGTAGCGTGGTTATTAAAAATTAGGGGAGTGTAGGCTGGAGCTGCTTC
PG513	TAGTTTTCTATGCAATTCGTTCAATAAAATTGTCTAAAATAACTATTACACATATGAATATCCTCCTTAG
Confirmation of deletion	
PG514	CACTATGTGAGTAGCTAAAT
PG515	ACGGTTGAAAATAGAGACGGT
ompA cloning	
PG492	CATATGGCTCCGAAAGATAACACCTG
PG493	CTCGAGAGCCTGCGGCTGAGTTACAAC

^a^ bases underlined correspond to the regions hybridizing to the pKD4 plasmid

### Expression and purification of *E*. *coli* recombinant OmpA

The rEcOmpA protein was produced as inclusion bodies and purified under denaturing conditions. Briefly, the ompA gene from strain *E*. *coli* P4 was amplified by PCR using primers PG492 and PG493 (Table 1) and cloned into the pET14b vector (Invitrogen) by NdeI-XhoI sucloning. The ompA sequence was verified by sequencing and the resulting plasmid was transformed in *E*. *coli* BL21(DE3). Expression of OmpA fused to a histidine tag was induced by growing bacteria in LB broth in the presence of 0.01 mM IPTG for 24 h at 37°C. Bacteria were then collected by centrifugation and purification was performed as described [16]. Briefly, bacteria were resuspended in phosphate buffer with 500 mM NaCl and 0.5 mM EDTA then lysed by sonication. The insoluble fraction was collected and resuspended in 8M urea and purified on Ni-NTA columns as described by the manufacturer (Qiagen). A stock solution of rEcOmpA (1 mg/mL in 4 M urea and 250 mM imidazole) was thus obtained and stored at -80°C. We did not try to refold the purified rEcOmpA. Purity of the rEcOmpA preparation was evaluated by SDS-PAGE followed by Coomassie blue staining. Western-blotting with an anti-ompA antibody (provided by Dr R. Lloubès) was also used to assess that the purified protein was *E*. *coli* OmpA.

The presence of LPS contamination in the rEcOmpA suspension was measured by incubating HEK-TLR4 cells (Invivogen) for 24 hours in the presence of different concentrations of the recombinant protein. rEcOmpA was also incubated with HEK-TLR2 reporter cell lines (Invivogen) to evaluate the TLR2-specific response. Control wells included stimulation with different concentrations of ultrapure LPS (ultrapure LPS-EB from *E*. *coli* O111:B4, Invivogen), a TLR4 agonist, or the TLR2 agonist Pam3-CSK4 (Invivogen). Responses of HEK-TLR2 and HEK-TLR4 cells were measured by quantifying human CXCL8 secreted in the supernatant by ELISA (Peprotech).

### Animals and experimental scheme

Healthy lactating cows in their first or second lactation were selected on the basis of low somatic cell counts (SCC) in udder quarter milk (range 6,000 to 128,000, mean 28,000 cells/mL) and absence of intramammary infection by major pathogens (*Staphylococcus aureus*, *Escherichia coli* or streptococci). The shedding of *Corynebacterium bovis* or coagulase-negative staphylococci in milk of some quarters was tolerated when not accompanied by clinical signs (clots in milk or altered mammary gland at visual or palpation examination). Udder quarters selected for intramammary challenge were free of infection.

Twenty cows were involved in the study. Cows were not separated from their herdmates during the experiment and they remained in the herd after the completion of the study. All cows were milked two times daily and were housed in a loose-housing cowshed. They were fed a diet of hay, silage and concentrate. Twelve cows were immunized with rEcOmpA. They received one intramuscular injection of 20 μg rEcOmpA followed by a booster injection of 10 μg rEcOmpA one month later, both in the prescapular region. The antigen was emulsified (water in oil emulsion) in Montanide™ ISA 61 G (Seppic, Puteaux, France) as described [8]. Eight cows, which received the adjuvant only, served as control animals. One immunized cow was inadvertently culled before the intramammary challenge.

The humoral and cellular responses were monitored by collecting a series of blood samplings by tail venipuncture, which were used to measure antibodies to rEcOmpA in blood serum and to perform whole blood antigen stimulations. The cows were then challenged by infusion of rEcOmpA into one udder quarter through the teat canal, and milk samples were collected during the 4-day follow-up period to monitor the local inflammatory response, essentially as described previously [8].

### Intramammary challenge with rEcOmpA

The challenge consisted in the instillation of rEcOmpA into one udder quarter through the teat canal after the morning milking. Purified rEcOmpA was diluted in cell culture grade Dulbecco’s phosphate buffered saline (Lonza, Verviers, Belgium) supplemented with 0.5 mg/mL pyrogen-free bovine serum albumin (cell culture grade, Sigma) just before use. Dilutions of rEcOmpA were calculated so that the tested dose was administered under a volume of 1 mL. Immediately after the morning milking, 1 mL of the freshly prepared rEcOmpA solution was infused through the teat canal by using a sterile smooth cannula fitted to a 1-mL disposable syringe. The challenge quarters were sampled with asepsis precaution just before infusion, at the following evening milking, at 14 h post-infusion (hpi), then at each milking for three more days. Bacteriological examination and SCC were carried out as described to check that the glands had not contracted an infection and to assess the local inflammatory response. Analgesics were not used because the cows did not display overt pain manifestations after challenge. Anti-inflammatory drugs were not administered since inflammation was the reaction under scrutiny.

Three distinct experiments were carried out. A pilot experiment was designed to determine the dose of rEcOmpA that would not induce an inflammatory reaction in unimmunized cows. Three unimmunized cows with at least three healthy uninfected quarters were challenged in three quarters, one with 1 μg, another with 3 μg and the third one with 9 μg rEcOmpA. The 1 μg and 3 μg doses did not induce any SCC increase, but one out of the three quarters infused with the 9 μg dose reacted with more than 1 million cells/mL milk at 8 hpi (results not shown). Accordingly, it was decided to use a 3 μg dose of rEcOmpA to challenge the immunized cows. The second experiment involved 6 immunized cows and 4 control cows, which received 3 μg rEcOmpA into one healthy quarter. As the induced responses were very limited, a second round of challenge was carried out with 9 μg of rEcOmpA.

### Assessment of humoral immune response by ELISA

To determine antibody titers, serum samples were collected by tail venipuncture at days 0, 30, 45 and 60 after the first immunization. Antibody responses to rEcOmpA and to whole *E*. *coli* were evaluated by using two distinct ELISAs. To determine rEcOmpA titers, ELISA plates (Nunc Immunosorp Maxisorp, 96 flat-bottom wells) were prepared by distributing 100 μl/well of rEcOmpA (1 μg/ml) in phosphate buffered saline (PBS) in the wells. After an overnight incubation at 4°C, the plates were emptied and blocked with 200 μl/well of gelatin (5 mg/ml) for 1 h at 37°C. The plates were washed once with deionized water, and serum samples at appropriate dilutions were incubated for one hour at 37°C. After three washes with PBS supplemented with 0.05% Tween 20 (PBS-T), antibody binding was detected with anti-bovine IgG (H+L) horseradish peroxidase (HRP)-conjugated secondary antibodies (Jackson Immunoresearch Inc., West Grove, PA, USA). Titration was carried out by incorporating in each plate a series of twofold dilutions of a reference serum from a cow immunized with rEcOmpA (cow #5, collected at D60), which was given an arbitrary titer of 1000 Units/ml. This serum yielded an OD_620_ of 1.17 at the 1/1000 dilution and 0.46 at the 1/5000 dilution when the stop solution was added after about 5 min of substrate incubation. Individual sera under test were given a titer by comparison with the obtained standard curve using the software operating the ELISA reader (Genesis Lite, Life Sciences, UK).

To titrate antibodies against whole *E*. *coli* bacteria, ELISA plates were coated with heat-killed (60°C for 30 min) *E*. *coli* P4 or the mutant P4Δrfb. Prior to incubation with bacteria, ELISA plates (Nunc Immunosorp Maxisorp, 96 flat-bottom wells) were prepared by distributing 100 μl/well of poly-L-Lysine at 1 μg/mL in PBS and incubation for 2 h at ambient temperature. Then, bacterial suspensions (OD_620_ = 0.2 in PBS, 100 μl/well) were incubated overnight at 4°C, most of the PBS removed and the plates were dried by incubation for 24 h at 37°C. Antibody binding was detected with the anti-bovine IgG (H+L) HRP-conjugated secondary antibody (Jackson Immunoresearch Inc). Antibody titration was performed with reference to a pool of sera from 16 unimmunized cows of the herd (LEGTA, Vendôme), considered representative of the titer of natural antibodies to *E*. *coli*.

For the isotype-specific ELISA detecting antibodies to the mutant P4Δrfb, sheep anti-bovine IgG1 or anti-IgG2, conjugated to horseradish peroxidase were used (AbD Serotec BioRad). Because of the scarcity of IgG antibodies in the pooled serum, the immune serum was used as for the rEcOmpA ELISA, and was attributed 1000 units of IgG1 and IgG2 antibodies.

### Assessment of the accessibility of OmpA to antibodies

To assess the accessibility of OmpA to antibodies, we used the ELISA with P4 or P4Δrfb strains as coating antigen and OmpA-specific antibodies. For anti-OmpA antibody preparation, purified rEcOmpA was sent to Eurogentec (Belgium) and used to raise antibodies in rabbits using their speedy 28-day polyclonal antibody protocol. This protocol involved the injection of 0.1 mg of protein into each of two rabbits at days 0, 7, 10, and 18 and a final bleeding at day 28. The final immune serum was used to prepare affinity-purified antibodies. To this end, the immune serum was heat-treated at 56°C for 30 min to inactivate the complement and passed through a column of rEcOmpA immobilized on Ni-NTA gel, equilibrated in 50 mM Tris pH 7.4 plus 0.15 M NaCl. The column was washed with Tris buffer supplemented with 1.0 M NaCl, and antibodies were eluted with 3.0 M MgCl2, 0.075 M HEPES with 25% ethylene glycol pH 7.2 [17]. The eluted fraction was desalted by exclusion chromatography on PD10 columns (GE Healthcare) equilibrated in Dulbecco’s PBS and stored in fractions with or without 50% glycerol at -20°C. An antiserum to whole P4 wt bacteria was also prepared by immunizing a rabbit with 2 x 10^9^ heat-killed bacteria subcutaneously in a water-in-oil emulsion.

### Assessment of cellular immune response to immunization

Whole blood antigenic stimulation (WBA) was performed as described [8] with some modifications. Blood samples (100 μL) were incubated in triplicate with 100 μL of either culture medium (RPMI-1640 supplemented with 10% fetal bovine serum, 2 mM L-glutamine, 10 mM HEPES, penicillin-streptomycin, fungizone and 0.05 mM β-2-mercaptoethanol) as baseline control, pokeweed mitogen (2 μg/mL) as positive control, or rEcOmpA (20 μg/mL) in 96-well tissue-culture microplates. At the end of the 48 h-incubation, the supernatant was collected and concentrations of IL-17A and IFN-γ were determined by ELISA [8]. Results were corrected for the baseline production of cytokines in the assay by subtracting the ELISA values of unstimulated blood from values of rEcOmpA stimulated blood. To ascertain that the production of IL-17A and IFN-γ was dependent on CD4+ T cells, peripheral blood mononuclear cells (PBMC) were isolated from two immunized cows and depleted of CD4+ cells by using MACS® beads (Miltenyi Biotech, Bergish Gladbach, Germany) as described [8]. Percentages of CD4+ T cells were about 12% before and less than 1% after depletion, as assessed by flow cytometry as described [8]. The antigen-specific stimulation assay was performed on depleted and unfractionated PBMC.

### Statistical analysis of data

The distribution of tested variables was assumed non-normal. Accordingly, non-parametric statistical analyses were used with the occasional exception: the kinetics of the milk leukocytosis response was analysed with the ANOVA for repeated measures with Tukey’s correction. Descriptive univariate analysis was performed through unadjusted Kruskal-Wallis test and further post-hoc Tukey test for pairwise multiple comparisons. Correlation analyses were done with the Spearman’s rank order test. All analyses were performed with either the GraphPad Prism 7.03 or the XLSTAT (XLSTAT-Base, Addinsoft) softwares. Significance was declared at *p* < 0.05 for all analyses.

## Results

### Expression and purification of rEcOmpA

**Fig 1:**
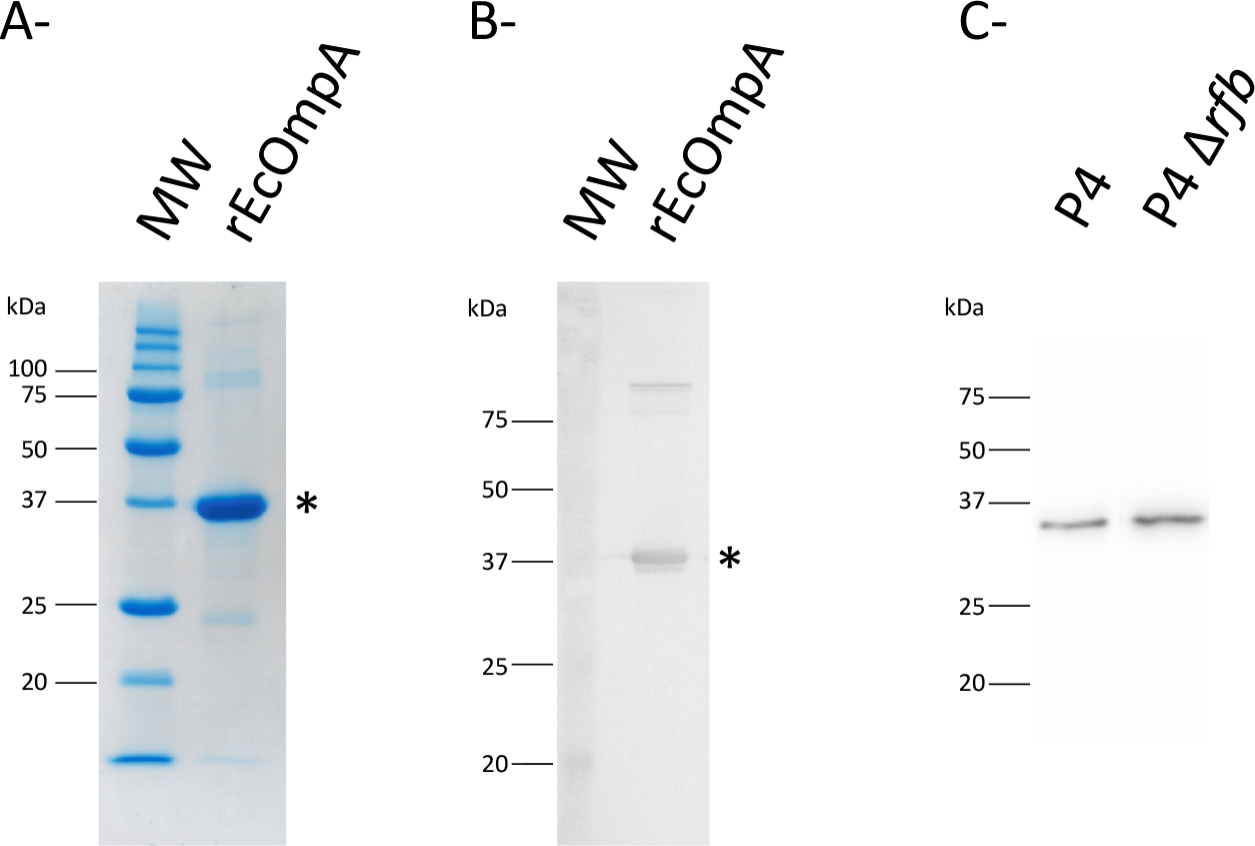
Assessment of rEcOmpA purity. 5 μg of rEcOmpA were analysed by SDS-PAGE and Coomassie blue staining (A) and by western-blotting with a rabbit anti-ompA antibody (B). Bacterial pellets from the wt and Δrfb P4 strains were immunoblotted with rabbit antibodies to OmpA, showing that similar amounts of OmpA were produced by both strains (C).

The purified rEcOmpA was evaluated by SDS-PAGE and western-blotting with an anti-OmpA antibody (Fig 1). To assess the presence of LPS in the purified preparation, we exposed HEK cells expressing the TLR4 receptor that recognizes LPS to different concentrations of rEcOmpA. Stimulation of HEK-TLR4 cells with rEcOmpA at 100 μg/mL led to secretion of CXCL8 to a level in between those obtained upon stimulation with uLPS at 10 ng/mL and 1 ng/mL. Stimulation of HEK-TLR2 cells with rEcOmpA at a concentration of 100 μg/mL resulted in a response intermediate between that obtained with 1 and 10 ng/mL Pam3-CSK4.

### Antibody response to recombinant EcOmpA

**Fig 2:**
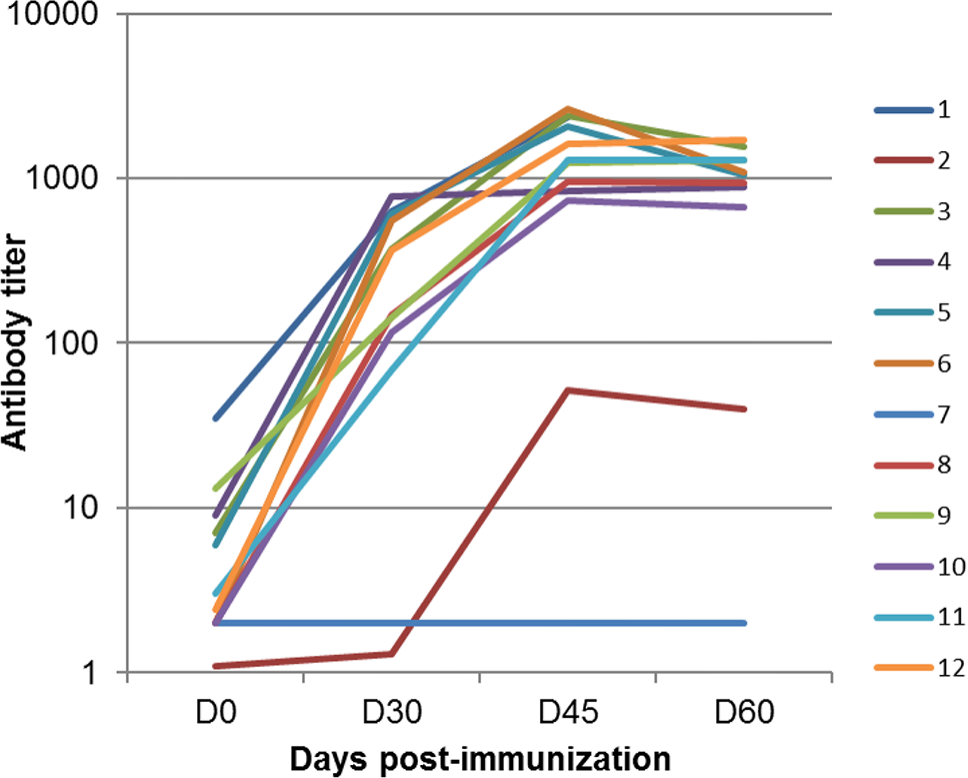
Humoral response to immunization with rEcOmpA. Cows were immunized on days 0 and 30, and the antibody response was measured by ELISA using rEcOmpA as antigen. Titers are expressed with reference to the serum of one immunized cow given an arbitrary titer of 1000 units. Antibodies were detected with a secondary antibody which reveals both IgG and IgM isotypes.

Immunization with rEcOmpA elicited a marked increase in antibody titers, with a 2-log average, in 10 out of the 12 cows (Fig 2). One cow did not respond, another responded poorly, both had very low initial antibody titers (Fig 2). To determine if antibodies recognizing the portion of OmpA which is exposed in its native form at the surface of the outer membrane were elicited, we performed the ELISA by using whole bacteria as coating antigen.

**Fig 3:**
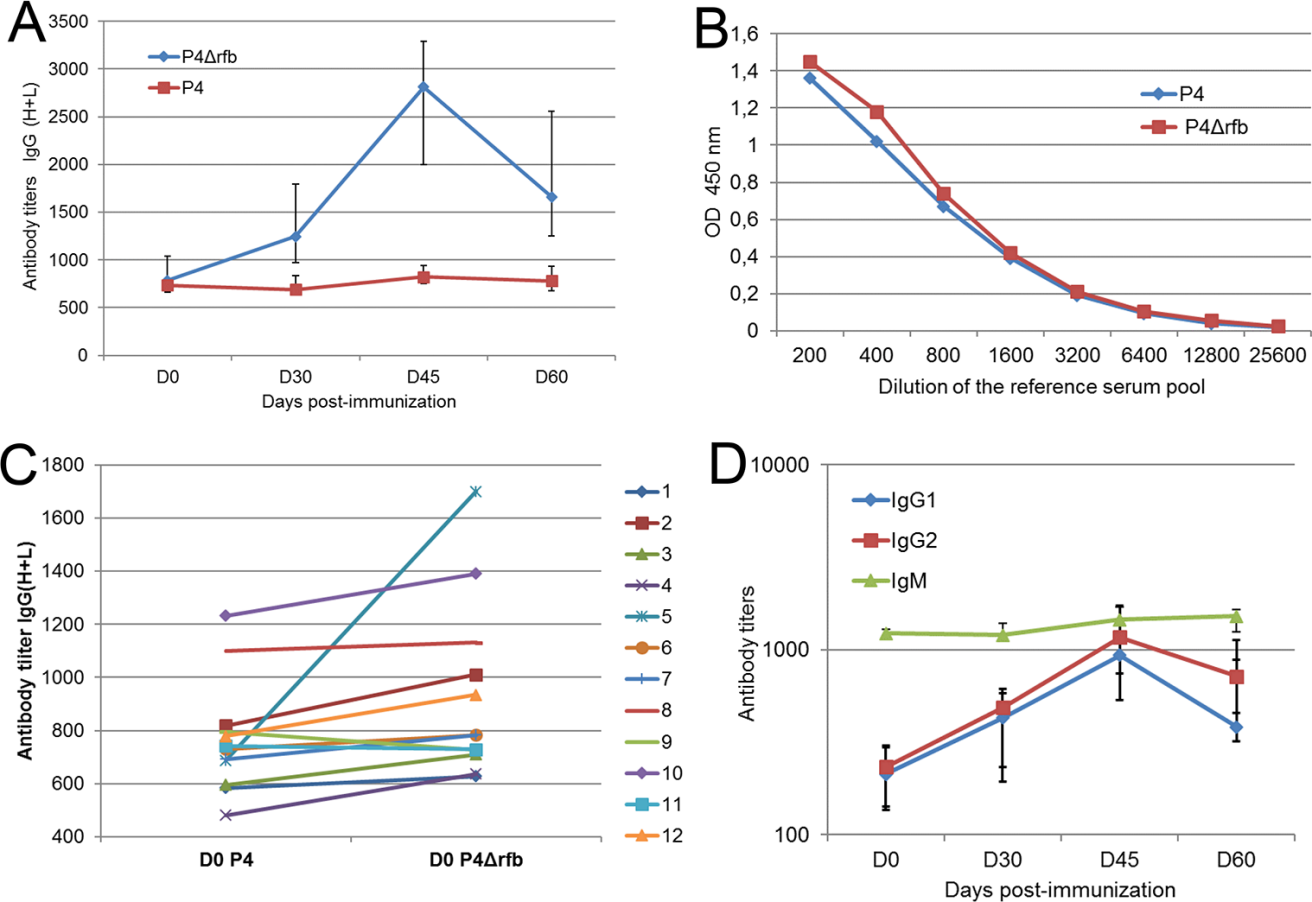
Humoral response to immunization with rEcOmpA. A) The response to immunization with rEcOmpA (on days 0 and 30) was assessed by ELISA using whole heat-killed smooth (P4) and rough (P4Δrfb) bacteria as coating antigen. Data are median values (12 immunized cows) and interquartile (Q1 and Q3). B) Reference curve of the pooled serum in ELISA with *E*. *coli* P4 and P4Δrfb as coating antigens and secondary antibody to bovine IgG(H+L) revealing IgG and IgM antibodies. C) Titration of natural antibodies detected before immunization, showing the correlation between titers of antibodies to *E*. *coli* P4 and P4Δrfb. D) Titration of antibodies to *E*. *coli* P4Δrfb in the IgM isotype and IgG1 and IgG2 subisotypes. As different reference sera, and different secondary antibodies were used for IgM and IgG antibodies, only titers variation can be compared, not their absolute values.

When the antibody content of the sera was assessed by using P4 bacteria as the ELISA coating antigen, there was no detectable increase in titers following immunization (Fig 3A). Suspecting that the LPS coat shielded the outer membrane protein OmpA, we prepared a rough mutant of strain P4. When used as ELISA antigen, the rough bacteria (P4Δrfb) revealed an increase in antibody titers following immunization (Fig 3A). The increase in antibody titers to P4Δrfb following immunization with rEcOmpA indicates that at least part of the induced antibodies were able to recognize epitopes of the native antigen exposed at the external face of the outer membrane. The difference in reactivity of antibodies to EcP4 and EcP4Δrfb is in favor of the hypothesis of outer membrane shielding by complete LPS.

Interestingly, the pooled bovine serum reacted to both bacteria with very similar magnitude and average avidity, as shown by the almost identical titration curve as a function of dilution (Fig 3B). This made it possible to compare the antibody titers obtained with the two antigens. Of note, all tested cows possessed naturally acquired antibodies to *E*. *coli* P4 and P4Δrfb before immunization, and the titers to the smooth and rough bacteria were correlated (Spearman’s r = 0.643, p < 0.025; Fig 3C). Consequently, these animals cannot be considered naïve to *E*. *coli* P4, and the amount of naturally acquired antibodies to the smooth and rough bacteria seems to be related.

As natural antibodies are usually mainly of the IgM isotype, the P4Δrfb ELISA distinguishing the IgM and IgG isotypes and sub-isotypes was carried out. To titrate IgM antibodies, the pooled normal serum with 1000 attributed antibody units was used. When the IgG1 and IgG2 antibodies were titrated, it appeared that the pool of normal serum could not be used as standard, because of a very low ELISA signal. The immune serum (of cow #5, immunized with rEcOmpA) was used instead, with arbitrary titers of 1000 units of IgG1 and of IgG2 antibodies. This indicated that most natural antibodies were of the IgM isotype. Antibody titers hardly increased in the IgM isotype following immunization (Fig 3D). By contrast, antibody titers augmented by a factor 4 in the IgG_1_ and IgG_2_ isotypes (Fig 3D).

### Accessibility of OmpA at the surface of *E*. *coli* P4

**Fig 4:**
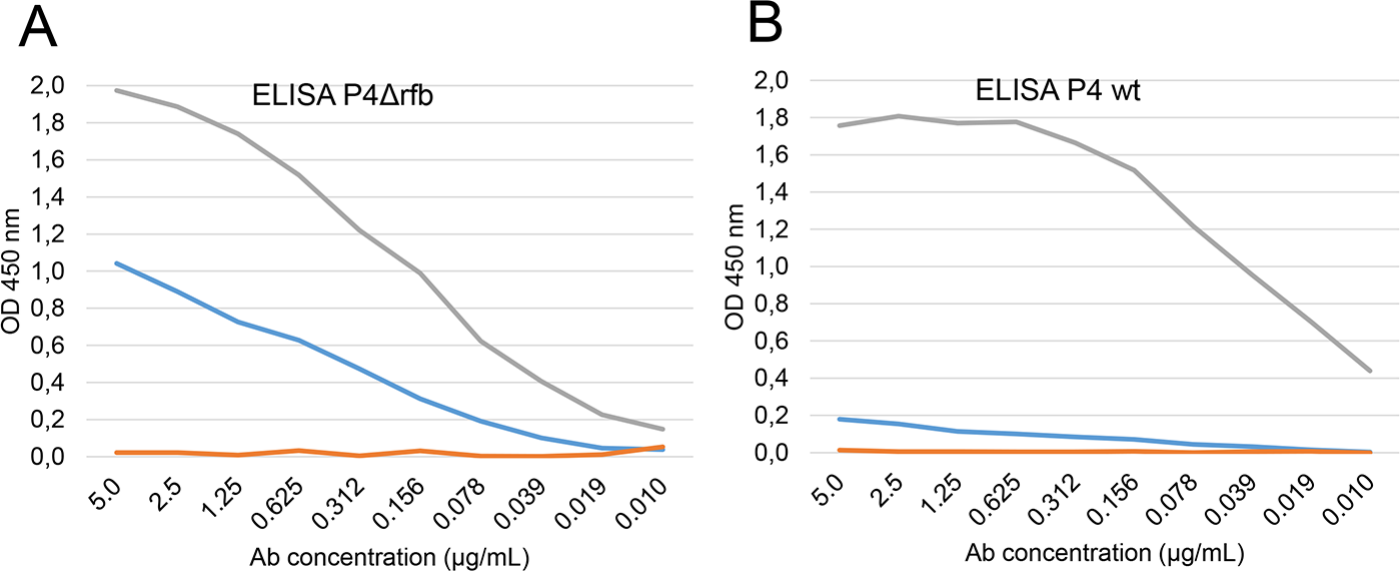
Reactivity of antibodies to OmpA with whole bacteria. Twofold dilutions of affinity-purified antibodies to rEcOmpA (blue lines) or to ovalbumin (red lines) from 5 μg/mL onwards were incubated with either P4Δrfb (A) or P4 wt bacteria (B) as ELISA antigen. Twofold dilutions (from 1/100 onwards) of a rabbit serum to P4 wt bacteria was used as a positive control (grey lines). Data are means from 3 experiments.

To investigate the issue of the accessibility of OmpA to antibodies more specifically, we prepared OmpA-specific antibodies raised in rabbit and purified by affinity on rEcOmpA. The affinity-purified antibodies reacted markedly with the P4Δrfb strain, but poorly with the parent P4 strain (Fig 4). The positive (antiserum to whole P4) and negative (affinity-purified antibodies to ovalbumin) controls indicated that the ELISA was able to detect specifically antibodies to OmpA (Fig 4). To check that the high reactivity of the P4Δrfb strain was not due to an overexpression of OmpA, we compared the production of OmpA by the wt and the mutated P4 strains. After an overnight culture in LB broth, bacteria were washed and bacterial suspensions adjusted to the same cfu numbers. Bacterial pellets were resuspended in loading buffer, boiled for 5 min, submitted to SDS-Page and immunoblotted with Accessibility of OmpA rabbit anti-OmpA antibodies. Similar bands were obtained with the wt and mutant strains, ruling out the overexpression possibility (Fig 1C). These results are in keeping with a limited accessibility of OmpA for antibodies at the surface of strain P4.

### Cellular response to rEcOmpA

**Fig 5:**
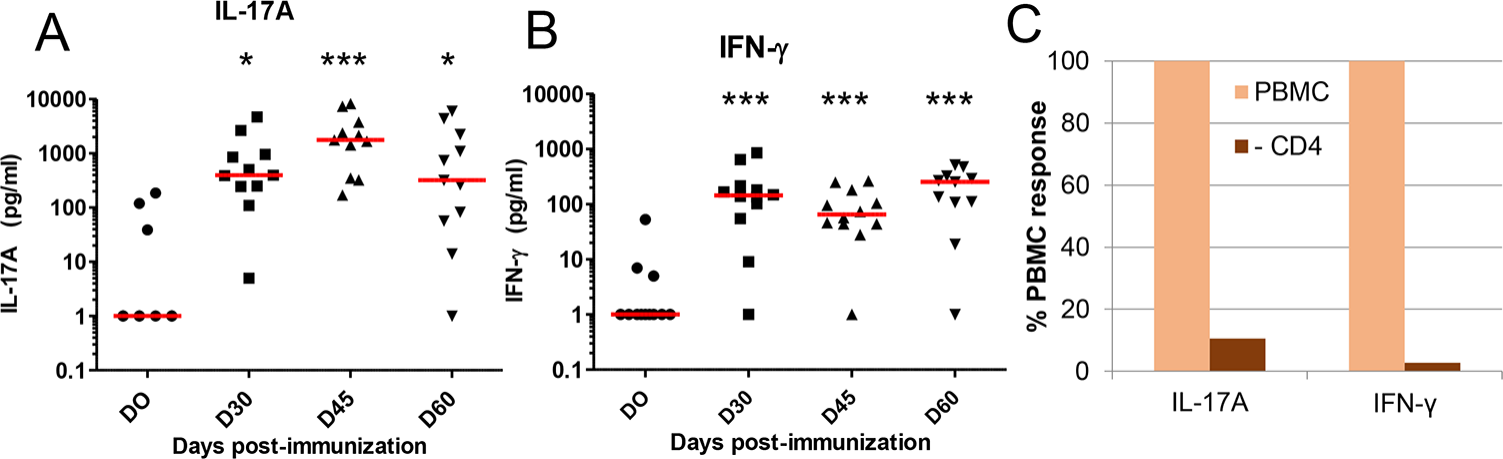
Production of IL-17A and IFN-γ by blood cells stimulated with OmpA. A) Time-course production of IL-17A in the OmpA-specific whole blood assay. The blood of the 12 immunized cows was cultured in the presence of OmpA and the concentrations of IL-17A measured by ELISA. B) Time-course production of IFN-γ in the OmpA-specific whole blood assay. C) The effect of magnetic depletion of CD4+ cells from PBMC of two immunized cows on the production of IL-17A and IFN-γ. Means are expressed as the percentage of cytokine production by CD4-depeleted cells relative to the production by untreated PBMC (100%). Friedman test: p< 0.001 for IFN-g, followed by Dunn’s multiple comparison test versus D0, at D30 and D60 (ns at D45); IL -17A: Friedman test p < 0.0001, Dunn’s multiple comparison test: versus D0: D30 * D45 *** D60.

The cellular response to rEcOmpA was monitored by using the WBA with rEcOMpA as antigen. Before immunization, three cows produced IL-17A or IFN-γ, but only in low amounts (Fig 5A and 5B). Following immunization, productions of IL-17A and IFN-γ increased, with a peak 15 days after the booster injection for IL-17A, whereas for IFN-γ the production was at its highest 30 days after the booster. Overall, the IFN-γ response was weaker than the IL-17A response, in particular 45 days post-immunization, with median values of 1957 pg/mL for IL-17A and 65 pg/mL for IFN-γ. Nevertheless, even for IL-17A, only four cows responded markedly (Fig 5A).

To check whether the production of IL-17A and IFN-γ was related to the presence of CD4+ T cells in the assay, PBMC either depleted of CD4+ T cells or untreated were cultured with rEcOmpA for 48 h and cytokine concentrations were measured in the supernatants. Depletion of CD4+ T cells almost abolished the production of IL-17A and INF-γ, showing that the assay revealed the presence of antigen-specific CD4+ T cells (Fig 5C).

### Induction of mammary antigen-specific inflammatory response

**Fig 6:**
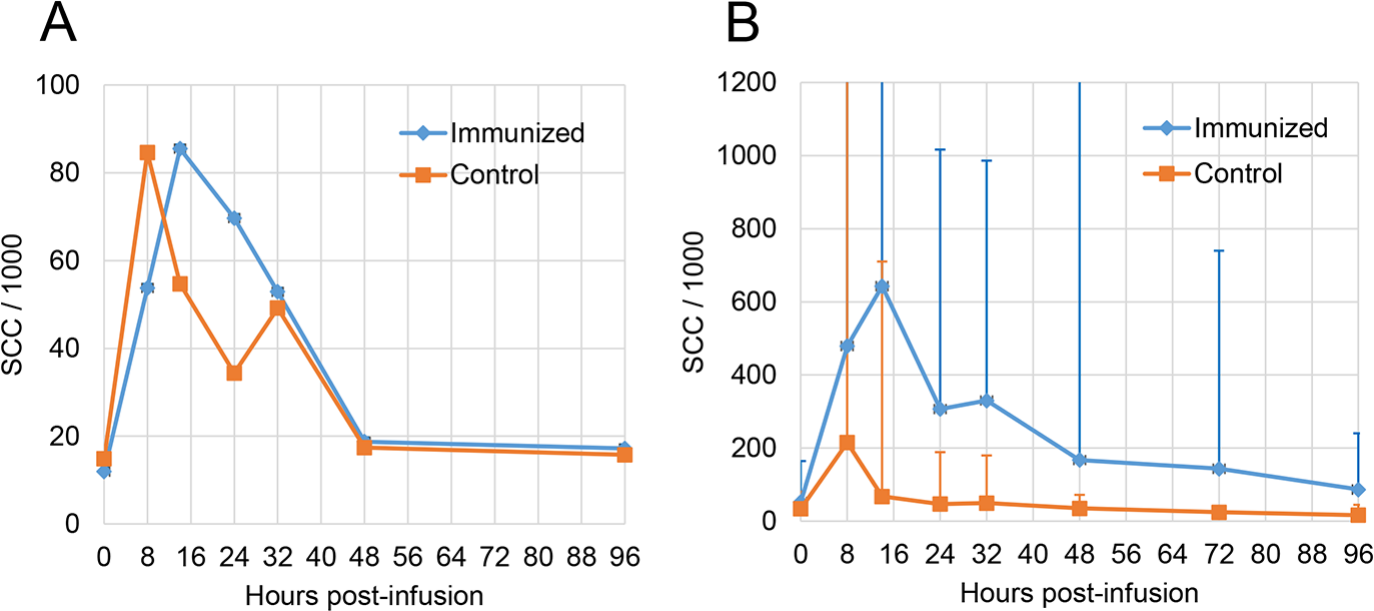
Mammary antigen-specific inflammatory response. A) Time-course of cell concentrations in milk (SCC) after infusion of 3 μg rEcOmpA through the teat canal of immunized and control cows. B) Time-course of cell concentrations in milk (SCC) after infusion of 9 μg rEcOmpA through the teat canal of immunized and control cows. Data are median values and interquartile (Q1; Q3).

A first round of intraluminal injection of 3 μg rEcOmpA was carried out involving five immunized and four control cows. All cows but one (control unimmunized) reacted with a moderate milk leukocytosis, albeit to different degrees. The main difference between the two groups of cows was the somewhat delayed response of the immunized cows with a peak SCC at 24 h post-challenge compared to 8 h post-challenge for the control cows (Fig 6A). Owing to the very moderate milk leukocytosis, the second round of challenge, involving six immunized and four control cows, was carried out with the dose of 9 μg rEcOmpA. All immunized cows but one, and two of the four control cows reacted to some extent. The magnitude of the milk leukocytosis tended to be higher in the immunized group over the observation period, the reaction was protracted in the immunized group, with a peak at 24 h post-challenge compared to 8 h post-challenge for the control group (Fig 6B). Notwithstanding, the difference was not significant (Kruskal-Wallis test or ANOVA for repeated measures).

### Correlations between humoral and cellular responses

There was a significant correlation between antibody titers to rEcOmpA and the IL-17A WBA results at the peak of the response (day 45 postimmunization), but not with the IFN-γ WBA (Table 2). Considering the antibody titers to *E*. *coli* P4Δrfb, representative of the humoral response to native and surface epitopes of OmpA, there was also a high significant correlation with the IL-17A WBA and a lower correlation with the IFN-γ WBA. There was no correlation between the antigen-specific mammary inflammatory response (SCC at 24 hpi) and the D45 IL-17A and IFN-γ WBA (Table 2). The better correlations of the WBA with the antibody titers to P4Δrfb than with antibody titers to rEcOmpA might indicate that the antibodies to the native epitopes recognized at the surface of P4Δrfb were more T–dependent than the other induced antibodies to rEcOmpA.

**Table 2:** Correlations between humoral and cellular responses to rEcOmpA.

	Ab to rEcOmpA D45	Ab to P4Δrfb D45	SCC 24 hpi
WBA IL-17A D45	0.776***	0.769***	0.200
WBA IL-17A D60	0.327	0.600*	0.025
WBA IFN-γ D45	0.235	0.508*	-0.309

Spearman’s rank test, calculated from the 12 immunized cows for the antibody (Ab) correlations, and from the 19 challenged cows for the SCC correlations.

* p < 0.05

*** p < 0.005

## Discussion

In this study we aimed at evaluating the immunogenicity of OmpA through the capacity of rEcOmpA to elicit antibodies and cell-mediated immune responses in the cow. The immunogenicity of *in vivo*-expressed and surface-exposed proteins is an important prerequisite to the development of an effective vaccine. As OmpA has been shown to skew the immune response in particular settings, such as vaccination in mouse models or *in vitro* stimulation of antigen presenting cells [4, 18], we also assessed the capacity of rEcOmpA to orient T cell-dependent immune responses. We found that rEcOmpA induced humoral and cell-mediated immune responses, but with limited functionality.

Immunization with rEcOmpA induced a two-log increase in antibody titers in a majority of the cows under experiments (Fig 3). This result established that rEcOmpA is a good immunogen to induce antibodies in cows. We used an ELISA with whole bacteria as antigen to assess if these antibodies were able to react with the native antigen, and more specifically to the surface-exposed part of OmpA. As OmpA is reported to be present at high copy number in the outer membrane [2], it is likely that if the Ab elicited with the rEcOmpA were able to interact with OmpA, titer increases would be detected by the whole bacteria ELISA. Unexpectedly, there was no increase in antibody titers against P4 bacteria following immunization (Fig 4A). This could have resulted from the preparation of the bacteria used to coat the ELISA plates, such as the growth conditions and the mode of killing. We considered that heat-treatment of bacteria (60°C for 30 min) would not alter the antigenicity of OmpA, because OmpA is a sturdy molecule which withstands heat treatment up to 70°C [19, 20]. To keep the native properties of the polysaccharide coat of the bacteria, the heat-treatment was limited to what was sufficient to kill the bacteria (30 min at 60°C). Also, microscope examination of the plates showed that bacteria were readily immobilized at the bottom of the wells, showing a dense bacterial lawn. An alternative explanation is that the antibodies could not reach their antigen target because of the O-antigen polysaccharide sidechain of LPS which constitutes a dense layer of hydrophilic neutral material [21]. This possibility was supported by the increase in titers obtained when bacteria devoid of the O side chain (P4Δrfb) were used as the ELISA antigen. The positive reaction indicated that at least part of the elicited antibodies to rEcOmpA were able to recognize surface-exposed OmpA epitopes, likely the surface-exposed loops of OmpA, and suggested that the O polysaccharide chains of P4 LPS prevented antibodies from reaching their target. To check that the absence of titer increases against P4 bacteria was really an absence of reactivity of antibodies to OmpA, we used affinity-purified antibodies to OmpA. These antibodies reacted with bacteria devoid of O antigen, but very little with the parental strain (Fig 5).

It has been shown that *E*. *coli* OmpA is accessible to proteases and is thus exposed at the surface of strain K-12 [22], which is a rough strain. Accordingly, we found that OmpA was accessible to antibodies at the surface of the rough mutant P4Δrfb. OmpA of *Mannheimia haemolytica* has been shown to be accessible to antibodies [23], but the issue of the accessibility of OmpA to antibodies at the surface of smooth *E*. *coli* strains has not been extensively investigated. Immunization with rEcOmpA elicited mouse antisera that were able to improve the phagocytosis of a strain of *E*. *coli*, suggesting that antibodies to OmpA were able to reach their target [5]. In the context of mastitis, this issue deserves scrutiny because the opsonic activity of antibodies is one if not the most important of their relevant biologic activities. In the case of OmpA, our study indicated that antibodies were induced in the opsonic IgG2 subclass, but that they are most likely to be ineffective as they cannot reach their antigen target.

We found little Ab to rEcOmpA in pre-immunization sera but sizeable amount of antibodies to P4 or P4Δrfb. One possible origin of natural antibodies to *E*. *coli* is the induction of cross-reactive antibodies by Gram-negative bacteria from the gut microbiota [24]. Bovine serum contains naturally acquired antibodies to *E*. *coli* [25, 26]. We found that most natural antibodies were in the IgM class, a result in keeping with previous observations [26, 27]. The amplitude of antibody titer increases in the IgG1 and IgG2 isoltypes was much less than the 2-log increase in titers detected with the rEcOmpA ELISA (Figs 3 and 4). This suggests that i) most natural antibodies to P4Δrfb contributing to the initial titer (D0) were directed to antigens other than OmpA, such as other outer membrane proteins; ii) some of the elicited antibodies were directed against epitopes not present on the native protein; iii) part of the antibodies induced by rEcOmpA were not directed to the epitopes of OmpA exposed at the outer leaflet of the outer membrane, as the C-terminal domain which can be immunodominant but located in the periplasmic space [28].

Immunization with rEcOmpA induced a CD4+ T cell-dependent production of IL-17A and IFN-γ in the WBA (Fig 6). Before immunization, cytokine production was very low or undetectable. The response to immunization was biased towards the production of IL-17A. As a T-cell immunogen, OmpA seemed to skew the response towards a Th17 response with a poor IFN-γ component. Previous studies with a model antigen (ovalbumin) but the same adjuvant and immunization schedule as used in the present study showed that mammary antigen-specific inflammation correlated with a marked Th1/Th17 immune response [8, 10]. *Salmonella* OmpA proved to be immunostimulatory as demonstrated by stimulation of IFN-γ production and enhanced expression of MHC and co-stimulatory molecules in mouse dendritic cells, thus indicating a Th1-polarizing capacity [29]. Another study suggested a Th2 polarizing activity of *Pasteurella multocida* OmpA in mice [18]. The divergent immune response polarizations may be accounted for by different bacteria and hosts. Alternatively, the immune response to OmpA could have been constrained by a pre-existing immune response, as cows were not naïve to *E*. *coli* and to OmpA as revealed by pre-immunization antibody titers. An immune deviation may have taken place [30]. There is increasing evidence indicating that commensal microbiota shape T cell homeostasis and induce memory T cell responses [31]. Supposing previous encounters with *E*. *coli* have left a memory T cell pool with a certain bias, the response to the subsequent immunization with OmpA might have been pre-determined to this pathway, precluding the development of Th17-IFN-γ cells.

Whatever the mechanism underlying the observed biased cell-mediated response, our study suggests that a T cell response, including the production of INF-γ in the WBA, is a necessary component of mammary gland sensitization. This is because the intramammary instillation of rEcOmpA did not induce a sizeable milk leukocytosis, contrary to the strong reaction induced with ovalbumin [8], and because the WBA did not correlate with the milk leukocytosis. Accordingly, the orientation of the response induced by rEcOmpA could reduce its capacity to trigger a mammary neutrophilic inflammatory reaction to infection.

Interestingly, the control unimmunized cows reacted to the intramammary instillation of rEcOMpA with a moderate milk leukocytosis. The presence of small amounts of LPS in the rEcOmpA preparation might have contributed to the reaction elicited by the intramammary infusion of 9 μg rEOmpA in a few unimmunized cows. Contamination of OmpA with LPS is difficult to avoid, in particular by the lipid A part of LPS, because LPS is tightly bound to porins [32]. However, the rEcOmpA used in our study was purified from inclusion bodies, not from the outer membrane, which is likely to result in lesser contamination with LPS. Furthermore, the estimated amount of contaminated LPS (9 ng) in the rEcOmpA 9 μg dose is hardly sufficient to induce a reaction. We have seen that the mammary response to low amounts of LPS (0.2 μg per quarter) is highly variable among cows, with some cows hardly reacting [10]. As the rEcOmpA hardly stimulated HEK-TLR2 cells at a concentration of 10 μg/mL, it seems likely that the slight inflammation observed in the quarters of control cows (Fig 6) was a consequence of a possible synergistic agonist activity of OmpA on different receptors of the innate immune system.

In conclusion, immunization with the purified rEcOmpA elicited a noticeable antibody response. Thus, OmpA is an immunogen to the cow with the ability to elicit antibodies in IgG1 and IgG2 sub-isotypes. Nevertheless, the elicited antibodies were not able to interact with whole P4 bacteria, strongly suggesting that they were not able to reach the accessible portions (extracellular loops) of OmpA molecules exposed at the outer leaflet of the external membrane of this *E*. *coli* strain. This would compromise their interest as opsonin or their capacity to interfere with the *E*. *coli* physiology. More generally, shielding by LPS might apply to other proteins embedded in the outer membrane. The shielding of OMPs, including OmpA, by LPS is likely to have important implications for the function of OMP-specific antibodies. As our study was limited to only one *E*. *coli* strain, this issue has yet to be investigated with a panel of strains representative of mastitis-associated *E*. *coli* before implications can be drawn in terms of usefulness of inducing antibodies to OmpA and maybe other OMPs by vaccination. Besides a humoral response, rEcOmpA induced a cell-mediated immune response that appeared to be biased towards a Th17 response with a low IFN-γ production. This bias may compromise the development of a mammary neutrophilic response upon antigen encounter. In the main, the interest of OmpA as a vaccine immunogen was not established. As our study used only one dose of antigen, one adjuvant, and one route of immunization, the biased immune response to OmpA has to be confirmed by further studies using different parameters.
